# Soil physicochemical gradient from headwaters to flooded riparian zones in the Falsino River, Eastern Amazonia

**DOI:** 10.7717/peerj.20882

**Published:** 2026-04-09

**Authors:** Liana Pereira Belúcio, Carlos Armando Reyes Flores, Leidiane Leão de Oliveira, Amanda Frederico Mortati, Daiane Batista Rodrigues, Gildo Vieira Feitoza, Thiago André, Jochen Schöngart, Maria Teresa Fernandez Piedade, Alan Cavalcanti da Cunha

**Affiliations:** 1 Postgraduate Program in Environmental Sciences, Federal University of Amapá, Macapá, Amapá, Brazil; 2 Postgraduate Program in Tropical Biodiversity, Federal University of Amapá, Macapá, Amapá, Brazil; 3 Civil Engineering Department, Federal University of Amapá, Macapá, Amapá, Brazil; 4 Institute of Biodiversity and Forests, Federal University of West Para, Santarém, Pará, Brazil; 5 University of Brasilia, Brasília, Federal District, Brazil; 6 Ecology, Monitoring and Sustainable Use of Wetlands (MAUA Research Group), National Institute of Amazon Research (INPA), Manaus, Amazonas, Brazil

**Keywords:** Edaphic-hydrological parameters, Flood pulses, Abiotic attributes, Conservation units, Amazon riparian zones, Hydrographic basin

## Abstract

This study investigates the edaphic-hydrological gradients of riparian zones in the Falsino River basin, Eastern Amazon. We defined 21 sampling units based on water body order (2nd to 4th) to capture edaphic and hydrological variation. Maximum river level values (MRL) were represented using limnimetric rulers as proxy of flood pulse intensity, and physicochemical analysis was performed on 378 soil samples. The topographic measurements by Global Positioning System (GPS) and Shuttle Radar Topography Mission (SRTM) quantified basin levels and the results were compared. Results reveal a clear spatial pattern, with MRL increasing with tributary order and decreasing elevation. Flood pulse intensity strongly influenced soil texture and acidity, while variables such as organic matter and phosphorus were weakly associated. The inverse relationship between elevation and MRL highlights the topographic control on hydrological dynamics, although elevation alone did not explain most soil variations. Both GPS and SRTM showed strong agreement, validating their use in low-relief Amazonian landscapes. Sample plot delimitation along the basin’s longitudinal profile facilitated the evaluation of abiotic attribute relationships. This research provides novel insights into how hydro-topographic interactions shape riparian soil properties, offering a practical framework for hydropedological assessments in remote tropical basins.

## Introduction

Global analyses indicate that natural flooded areas declined by 31% between 1970 and 2008, with losses likely even greater in seasonally inundated zones (Dixon et al., 2016). In Europe and North America, up to 90% of floodplains are considered functionally extinct, and similar degradation trends are accelerating in developing regions (Tockner & Stanford, 2002). Consequently, riparian zones rank among the most threatened ecosystems globally, with biodiversity indicators, such as algae, fish, birds, reptiles, amphibians, and mammals, declining by up to 76% since the 1970s (World Wildlife Fund (WWF), 2004, 2014; Cunha et al., 2013).

On a regional scale, the physical and chemical properties of the Amazon soils vary predictably along the pedogenic gradients (Quesada et al., 2010). These properties are influenced by long-term weathering, which alters nutrient availability and grain size distribution. As a result, the edaphic characteristics of Amazonian soils are closely linked to the nature of the parent material and its sedimentary history (Higgins et al., 2011; Tuomisto et al., 2019).

Due to the multiple geological origins across the Amazon Basin (Räsänen et al., 1992, 1995; Sombroek, 2000), the region’s soils are highly heterogeneous (Sanchez & Buol, 1974; Tuomisto et al., 2016; Flores, da Cunha & Cunha, 2022). Nutrient scarcity in these soils is linked to leaching processes acting on exposed sediments. These conditions result in highly weathered, nutrient-poor soils, with variation shaped by differences in parent material and geomorphic setting (Villar et al., 2022; Moore et al., 2022). The oldest surfaces are in the Precambrian Guyana and Brazilian shields, where nutrient-poor conditions dominate (Quesada et al., 2010; Mendonça et al., 2020; Lange et al., 2024).

Amazonian floodplains exhibit diverse soil types shaped by the long-term deposition of sediments carried by rivers and streams that originate in the Andean Shield (Nittrouer et al., 2021). In contrast, there are areas known as *igapós*, which are seasonally flooded by blackwater or clearwater rivers whose headwaters originate in the Guiana and Central Brazilian Shields (Junk et al., 2015; Wittmann et al., 2022). These rivers are rich in humic and acidic compounds but poor in suspended sediments, leading to nutrient-deficient soil conditions (Santos & Cunha, 2015; Rocha et al., 2020).

While the geomorphology and soil physical-chemical properties of Amazon soils are widely considered as key contributors to the composition of river waters (Gaillardet et al., 1997; Quesada et al., 2011; Ríos-Villamizar et al., 2020; Lima et al., 2022), less attention has been given to the reverse relationship, that is, how fluvial dynamics influence the development and composition of alluvial soils. Among these dynamics, the seasonal flood pulse stands out as a key factor of ecosystem processes in flooded areas of the Amazon (Fleischmann et al., 2023) and in floodplain systems globally (Tockner, Malard & Ward, 2000; Melack & Coe, 2021; Mosepele et al., 2022).

In large rivers such as the Amazon, the spatial distribution of tree species is strongly influenced by the duration and amplitude of seasonal flood (Householder et al., 2021), as well as by local soil texture and granulometry (Wittmann et al., 2022).

Hydrographic basins naturally present altitudinal (topographic) gradients, guiding surface water flow from elevated areas toward river channels (Habit et al., 2022; Amosova & Ilicheva, 2024). The elevation differences influence soil heterogeneity through surface erosion and exposure of parent material (Khodadadi et al., 2024). Also, the topographic position regulates the hydrological and edaphic gradients, which in turn affect soil physicochemical properties (Schietti et al., 2014; Seifu, Elias & Gebresamuel, 2020; Haile et al., 2024).

At broader spatial scales, topography and hydrological factors have been shown to influence the redistribution of soil nutrients and the alteration of chemical properties, particularly through processes of deposition and lateral water flow (Bauke et al., 2022; Yang et al., 2023). Understanding how topographic and hydrological processes interact across spatial scale is essential for predicting biogeochemical patterns and the functioning of riparian systems. It is also important to assess how these systems may respond to pressures from land use change and climate change (McGuire et al., 2014; Schneider et al., 2017; Hoppenreijs, Eckstein & Lind, 2022).

In the present study, we analyzed the relationships among topographic variation, flood pulse intensity (maximum level of surface water level in monitored plots) and edaphic gradients (physical-chemical and textural) along the longitudinal profile of riparian zones in the Falsino River watershed, Eastern Amazon. The basin is located within the Amapá National Forest (FLONA/AP), protected area in the state of Amapá/Brazil, and features soils derived primarily from reworked sediments and rocks, likely of Late Cretaceous origin (Irion, 1978; Quesada et al., 2010).

Despite the known influence of topography and hydrology on soil properties in upland forests and floodplains of the Amazon, little is known about how these factors interact to shape soil texture and nutrient dynamics in riparian zones. These ecotonal environments, where topographic and hydrological gradients converge, remain understudied despite their ecological importance. Addressing this gap is essential for predicting how riparian systems respond to environmental pressures and hydrological changes.

In this context, three hypotheses were proposed: (1) the flood pulse is influenced by topographic variation but also conditioned by the order of the watercourse; (2) the physical-chemical variation of the soil (edaphic) is explained by both topographic and hydrological gradients (flood pulse) and (3) the topographic levels measured with different methods correlate strongly, and thus their potential effects on soil properties do not depend on the method used.

The specific objectives were: (1) Statistically test the correlation between flood pulse and topographic elevation and stream order; (2) Statistically test how topography and flood pulse (intensity) correlate with physico-chemical soil parameters; (3) Test whether the correlation between edaphic parameters and topographic elevation depends on how the latter is quantified.

## Materials and Methods

### Study sites

**Figure 1:**
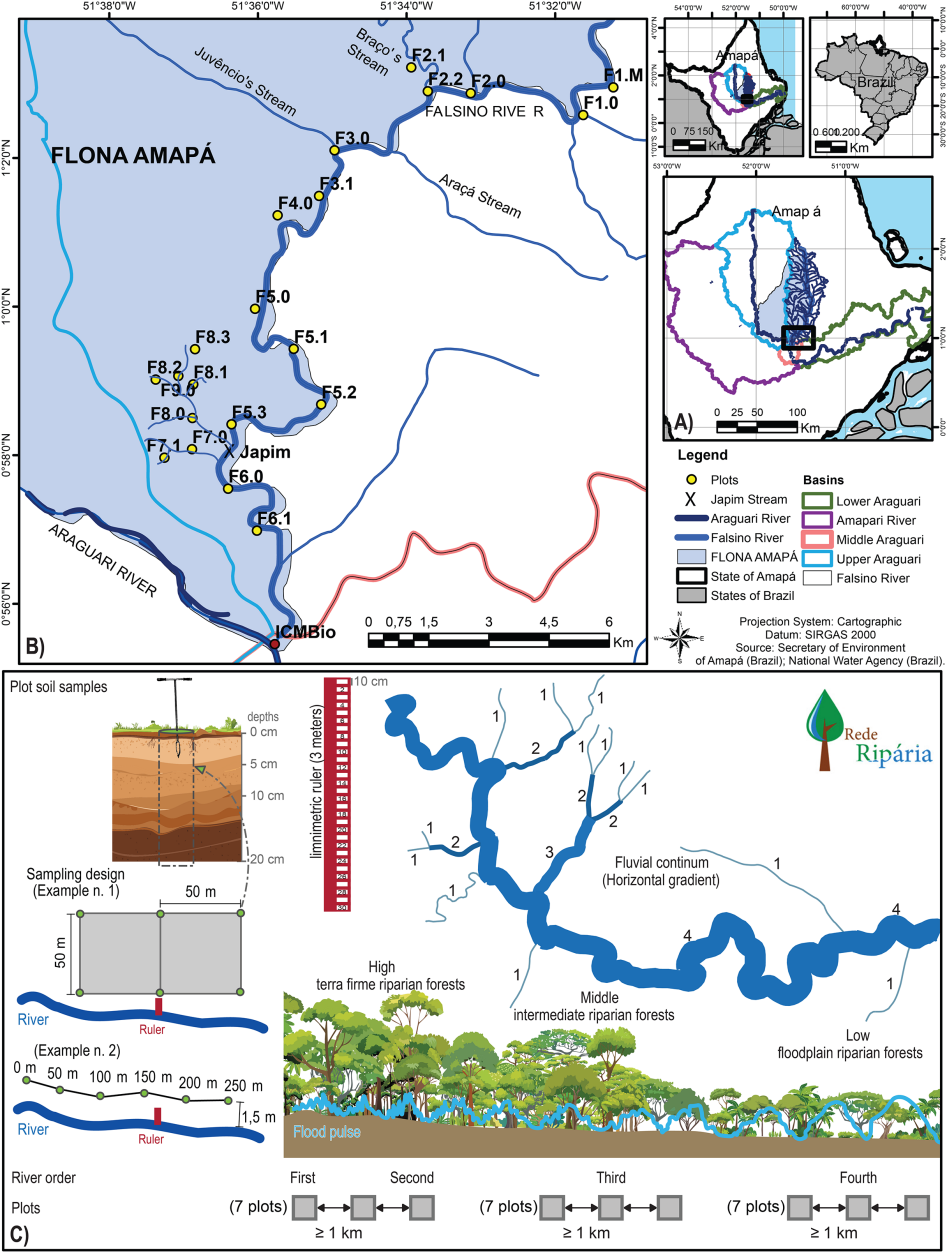
Spatial distribution and sampling design of riparian plots in the Amapá National Forest (FLONA) and Falsino River basin. (A) Location of the National Forest (FLONA) of Amapá, (B) drainage area of the Falsino River basin and distribution of riparian plots and (C) sampling design of the plot with limnimetric ruler and soil samples in two layouts, along the river in narrow areas without forest inventory, and in a rectangular format. The green dots indicate the sampling locations, and the soil profile diagram shows the depths of each subsample. The drawing in the upper right shows the numbers 1 through 4 along the drainage network that represent the stream orders of the tributaries. The schematic at the bottom of the figure highlights that there are seven plots within each of the three categories: Second-order streams; Third-order streams; and Fourth-order streams.

FLONA/AP, is located in the municipalities of Pracuúba, Ferreira Gomes, and Amapá (Fig. 1A). This federal conservation unit was created in 1989 and covers an approximate area of 459,867 ha (Fig. 1B). The climate is Hot-Humid Tropical (Af type, Köppen classification). According to Oliveira et al. (2020), the annual rainfall is 2,184 mm, and the internal precipitation on the soil below the canopy is 1,322.4 mm, which is distributed in two parts, one part forming the underground percolated and the other part forming the surface runoff in the forest.

The rainy season extends from December to July, and the dry season from August to November, with the warmest month being October and the coldest February. The average monthly temperature is defined between 25° and 26 °C, with monthly extremes reaching 32 °C (maximum) and 22 °C (minimum), based on data from the nearest municipality with reliable historical records (Oliveira et al., 2010).

The predominant soils in FLONA/AP are classified as Ferralsols according to the World Reference Base for Soil Resources (WRB), Oxisols according to the United States Department of Agriculture (USDA) Soil Taxonomy, and Red-Yellow Latosols according to the Brazilian Soil Classification System, commonly found in dense upland (“terra firme”) forest areas and associated with variable natural fertility. The area is covered mainly by dense ombrophilous forest with subdivisions into terra firme forest, *igapó* forest and some cerrado fragments.

The hydrographic network consists of the Rio Araguari basin, and it has two main tributaries, the Amapari and Falsino Rivers. These are perennial watercourses with seasonal flow variation, producing high discharge during the rainy months (600 m^3^/s, May 2019), and a significant drop of the flows from October to December (50 m^3^/s, August 2018) (De Souza et al., 2024).

The Araguari basin shows signs of environmental impacts, particularly in its middle and lower sections, where large hydropower plants and mining activities are present. Although the study area is located upstream within a protected area, signs of hydrological alteration have been observed near the mouth of the Falsino River, possibly related to backwater effects caused by downstream dam reservoirs.

### Sampling

Plot sampling units were established in a federal conservation unit classified and called the FLONA/AP (North: 51°30′25″W and 1°51′42″N; South: 51°35′41″W and 0°55′27″N; Least: 51°22′01″W and 1°24′44″N; West: 52°00′29″W and 1°11′07″N). Plots were installed in areas selected to minimize topographic variability, preserve habitat integrity, and maintain relatively constant relief conditions (Fig. 1A). In each plot, topography was considered uniform near the margins of surface water bodies.

Geometric factors of the basin were estimated in the drainage area, considered as relevant were: compactness coefficient of Gravelius (Kc), Form Factor (Kf) and drainage density (Dd) of the basin (Horton, 1945; Sassolas-Serrayet, Cattin & Ferry, 2018). These parameters were calculated based on area, perimeter and river length measurements obtained Google Earth software and georeferenced data from National Water Agency of Brazil (ANA, 2019).

The Gravelius compactness coefficient (Kc) was calculated based on the ratio between the basin perimeter and the circumference of a circle with the same area as the basin. The equation used is as follows:

(1) $$Kc = 0,\!28\; \displaystyle{P \over {\sqrt A }}$$

where *P* is the basin perimeter and *A* is the basin area.

The Form Factor (Kf) represents the ratio between the mean width and the axial length of the basin. The mean width is obtained by dividing the basin area (A) by its axial length (L). Thus, the Form Factor is calculated using the following equation:

(2) $$Kf = \displaystyle{A \over {{L^2}}}$$

where *A* is the basin area and *L* is the axial length of the basin.

The Drainage Density (Dd) indicates the drainage efficiency of the basin and is defined as the ratio between the total length *(∑l)* of all drainage channels and the total area *(A)* of the basin. It is expressed by the following equation:

(3) $$Dd = \displaystyle{{\sum l} \over A}$$

where *∑l* is the sum of the lengths of all stream segments and *A* is the basin area.

Soil and hydrological sampling units were defined considering the stream order, including second, third, and fourth order tributaries (Fig. 1C), to capture hydrological-environmental variation along the river continuum. Twenty-one samples were defined in the Falsino River watershed, from headwater to the main channel outlet (Fig. 1B, plots F1–F9). The plots, hereafter referred to as riparian plots, were spaced at a minimum distance of 1 km and positioned 1.5 m from the riverbank, where maximum river level (MRL) was quantified. The elevation data, for the correlation with MRL, was collected using Global Positioning System (GPS).

### MRL and soil physical-chemical properties

In each riparian plot, a limnimetric ruler was installed to measure the height of the water column. The limnimetric rulers were made of aluminum, 3 m high, and graduated every 10 cm (Fig. 1C). They were positioned at the bottom and the margin of the river channel of each plot. Monthly inspections were conducted over a 12-month period starting in August 2018. During peak flood periods (March to May), when water levels exceeded 3 m, additional rulers were positioned on top of the original ones to ensure complete measurements. The highest water column recorded in each plot during the monitoring period (12 months) was considered the maximum river level (MRL), used as a proxy for flood pulse.

Soil sampling in each plot was based on six subsamples taken once during the dry season (August) along a transect, spaced 50 m apart, always following the same topographic contour or positioned 1.5 m from the riverbank.

Collections followed similar designs whenever there was extensive flat terrain or sufficient space to distribute collection points within the plot. In these cases, a rectangular design was adopted, with defined length and width, to maintain collection points on the same topographic contour and capture the internal variation of the plot, whose total area is 0.5 ha. These plots were also delineated in this manner due to a forest inventory conducted for another research within the same project.

In the remaining plots, where there was not enough flat relief and forest inventory, the samples were distributed continuously along the streams or the main river, respecting the riverbank, totaling 250 m in length, with collection points positioned 1.5 m from the bank. Each soil subsample was collected at three depths (0–5, 5–10, and 10–20 cm) using a Dutch auger (Fig. 1C), following the protocols of the Biodiversity Research Program (PPBio, https://ppbio.inpa.gov.br/en/article_Biodiversity_Research_Brazil). For each plot, average values were calculated from all six subsamples and all three depths, after each one was analyzed in the laboratory. The subsamples were collected and analyzed separately due to other investigations that were required, but for this study, only the average values per plot were considered. The 0–20 cm interval was selected because nutrient concentrations in Amazonian soils are typically concentrated in this upper layer.

All soil samples were properly stored and sent for laboratory analysis of physical and chemical properties (Table 1), following the procedures outlined in the Manual of Soil Analysis Methods (Teixeira et al., 2017), and carried out at the Soil Laboratory of EMBRAPA (Brazilian Agricultural Research Company), Amapá, Brazil.

**Table 1:** Detailing the physicochemical analyses performed in soil samples from the Falsino basin (FLONA-AP).

Attributes		Abbreviation	Unit	Method/Soil extractant	Instrument
Physical	Particle size distribution (granulometry): Sand (fine, coarse, total), Clay, Silt	Fine_Sand, Coarse_Sand, Total_Sand, Clay, Silt	g/kg	Chemical and physical dispersion, sifting and sedimentation/Sodium hexametaphosphate, NaOH	Mechanical stirrer, sieve, oven, pipettor and analytical balance
	Soil Moisture	Moisture	(%)	Determination of water by difference in current and dry masses. It was measured once with the samples collected in the dry season.	Oven, analytical balance
Chemical	pH	pH	–	Determination of pH in soil sample dispersed in distilled water	Benchtop pH meter
	Organic Matter	Organic_Matter	g/kg	Spectrophotometry (visible, red filter, 630 nm)/Sodium dichromate in sulfuric acid solution	Horizontal circular shaker, analytical balance, UV-Vis spectrophotometer
	Available Phosphorus	P	mg/dm^3^	Spectrophotometry (visible, red filter, 660 nm)/Mehlich-1 extraction solution (HCl, H2SO4, H2O), ammonium molybdate in sulfuric acid solution	Horizontal circular shaker, analytical balance, UV-Vis Spectrophotometer
	Exchangeable Potassium	K	cmolc/dm^3^	Flame photometry/Mehlich-1	Digital Flame photometer
	Exchangeable Calcium and Magnesium	Ca_Mg	cmolc/dm^3^	Volumetric method by complexometric titration with EDTA/KCl extraction solution	Horizontal circular shaker, burette, analytical balance
	Exchangeable Aluminum	Al	cmolc/dm^3^	Volumetric method by titration/KCl extraction solution	Horizontal circular shaker, burette, analytical balance
	Potential Acidity	H_Al	cmolc/dm^3^	Volumetric method by titration/Calcium Acetate solution	Horizontal circular shaker, burette, analytical balance
	Sum of Non-Acid Cations	SNAC	cmolc/dm^3^	Sum of Ca^2+^ (Ca), Mg^2+^ (Mg), K^+^ (K)	–
	Total Cation Exchange Capacity pH7	CEC	cmolc/dm^3^	Sum of SNAC and H^+^ + Al^3+^ (H_Al)	–
	Saturation in Non-Acid Cations	Saturation_NAC	%	Percentage of SNAC in relation to CEC	–
	Aluminum Saturation	Al_Saturation	%	Percentage of Al^3+^ (Al) in relation to the sum of SNAC and Al^3+^	–

### Testing elevation measurement methods in edaphic correlations

To determine whether the correlation between edaphic parameters and topographic elevation depends on how elevation is determined, two elevation data sources were used: in-field measurements collected using GPS and remotely sensed data from the Shuttle Radar Topography Mission (SRTM). The SRTM provides digital elevation data with a spatial resolution of 90 m and is freely available for most regions of the globe.

Spearman’s rank correlation coefficients were calculated between each edaphic variable and elevation values derived from both GPS and SRTM sources. The resulting sets of correlation coefficients (R_GPS_ *vs*. R_SRTM_) were then compared using the Wilcoxon paired-sample test to assess whether significant differences existed between them (R values) across the 21 riparian plots.

This non-parametric test (Wilcoxon) was chosen because it does not require the assumption of normality, which is appropriate given the relatively small sample size and the likelihood that topographic elevation data in riparian environments deviate from a Gaussian distribution (Siegel & Wagner, 2022). In environmental studies, where data often violate parametric assumptions, this test is useful and reliable alternative for ensuring valid statistical inferences (Ramachandran & Tsokos, 2015).

### Data analysis

To identify patterns in soil physical and chemical properties across the riparian plots, a Principal Component Analysis (PCA) was applied. Previously, it was observed that many variables were collinear, such as total and coarse sand. The same for the sum of non-acid cations and calcium and magnesium (Ca_Mg), which are examples of parameters derived from calculations involving other variables. Due to high collinearity among edaphic variables, 10 of them were selected for the PCA, as representative indicators of particle size (granulometry or texture) and nutrient concentrations, excluding those that are strongly related to others. The variables analyzed were: Coarse sand, Clay, Silt, Soil Moisture, pH, Organic Matter, Available Phosphorus (P), Exchangeable Potassium (K), Exchangeable Calcium and Magnesium (Ca_Mg) and Exchangeable Aluminum (Al).

In addition to PCA, Spearman’s correlation test was used to examine relationships between all particle sizes (soil granulometry or texture), moisture, pH, and nutrient concentrations. The same test was also applied to assess correlations between riparian soil attributes, plot elevation, and maximum river level (MRL).

To test the topo-edaphic and hydrological parameters across stream orders (Ord2 = second-order rivers; Ord3 = third-order rivers; Ord4 = fourth-order rivers), appropriate non-parametric comparisons were performed.

All statistical analyses were conducted in R v. 3.6.2 (RStudio Team, 2020), using additional functions of the psych (Revelle, 2019) and car (Fox & Weisberg, 2019) packages.

## Results

### Descriptive analyses from Falsino basin

The Falsino Basin has an area of 4,199 km^2^ and a perimeter of 436 km, resulting in a compactness coefficient (Kc) of 1.89. This value indicates that the basin has an elongated shape, which implies a reduced ability to concentrate surface runoff flows simultaneously in the main drainage channel.

Basins with a high compactness coefficient tend to exhibit a more temporally distributed hydrological response. That is, the hydrological pulse generated by precipitation is propagated in a less concentrated manner, decreasing the likelihood of sharp peak flows at the outlet (Tucci & Hídricos, 2014; Reyes Flores, Ferreira Albuquerque Cunha & da Cunha, 2023; Sousa, Araújo & da Cunha, 2024).

The form factor (Kf) of the Rio Falsino watershed is 0.11, indicating an elongated shape. Watersheds with low form factor values tend to be less susceptible to flooding, as precipitation occurring in areas distant from the outlet takes longer to reach it, not contributing simultaneously to peak discharge. The drainage density (Dd) of the basin is 0.30 km/km², which, according to the classification by Tucci & Hídricos (2014), is indicative of poor drainage. This value reflects a sparsely developed drainage network, suggesting low efficiency in surface runoff. These morphometric parameters indicated that the Rio Falsino watershed exhibits characteristics that favor infiltration and delay runoff, thereby reducing the likelihood of flood events.

A descriptive and integrated statistical summary of the edaphic parameters is presented in Table 2 (average e standard deviation).

**Table 2:** Quantification of nutrients, moisture, organic matter and particle size distribution.

Attributes	Mean (all sampling sites and depths)	Standard deviation
Clay (g/kg)	151.29	88.44
Fine_Sand (g/kg)	286.44	190.19
Coarse_Sand (g/kg)	267.96	80.69
Total_Sand (g/kg)	554.40	189.47
Silt (g/kg)	283.87	131.85
Moisture (%)	26.40	7.64
pH	4.52	0.31
Organic_Matter (g/kg)	43.41	15.96
P (mg/dm^3^)	9.83	3.69
K (cmolc/dm^3^)	0.08	0.03
Ca_Mg (cmolc/dm^3^)	0.90	0.55
Al (cmolc/dm^3^)	0.97	0.33
H_Al (cmolc/dm^3^)	6.19	1.83
SNAC (cmolc/dm^3^)	0.98	0.57
CEC (cmolc/dm^3^)	7.36	2.11
Saturation_NAC (%)	14.34	4.86
Al_Saturation (%)	51.89	10.98

### Flood pulse in geographic and topographic gradients

Table 3 shows the existence of significant differences between the three river orders. These categories showed significant differences when comparing the maximum river levels (MRL) but showed no difference between the topographic (elevation) levels. Therefore, the flood pulse varied according to the order of the tributary. This is demonstrated through the Kruskal-Wallis test for the groups of plots categorized by river order, as well as the Spearman correlation test (Table 4). The result showed a strong positive correlation (r = 0.88, *p*-value < 0.0001). In Table 4, a moderate negative correlation was also observed between flood pulse and elevation (r = −0.47, *p*-value = 0.035). However, the order of the tributary and topography did not present a significant relationship (*p*-value = 0.1052).

**Table 3:** Kruskal-Wallis test for topo-edaphic and hydrological parameters, among different order tributaries. The fourth column includes significantly different tributary orders by Dunn’s test.

Variables	Kruskal-Wallis test (Ord2, Ord3, Ord4)*		Dunn’s test
Topographic-Hydrological	Χ^2^	*p*-value	Significant difference (Ord2, Ord3, Ord4) (*p* < 0.05)
Elevation by GPS (m)	2.71	0.2584	–
Maximum River Level (MRL) (cm)	14.99	0.0006	Ord2-Ord3; Ord2-Ord4
Edaphic variables	Χ^2^	*p*-value	
Al (cmolc/dm3)	9.14	0.0104	Ord2-Ord3; Ord2-Ord4
Al_Saturation (%)	10.97	0.0042	Ord2-Ord3; Ord2-Ord4
Saturation_NAC (%)	8.17	0.0168	Ord2-Ord3; Ord2-Ord4
Ca_Mg (cmolc/dm3)	8.24	0.0162	Ord2-Ord4
CEC (cmolc/dm3)	0.70	0.7033	–
H_Al (cmolc/dm3)	3.77	0.1516	–
K (cmolc/dm3)	4.59	0.1009	–
Organic_Matter (g/kg)	0.70	0.7033	–
P (mg/dm3)	2.56	0.2781	–
pH	4.88	0.0871	–
SNAC (cmolc/dm^3^)	9.28	0.0097	Ord2-Ord4
Moisture (%)	12.42	0.0020	Ord2-Ord4
Fine_Sand (g/kg)	1.16	0.5605	–
Coarse_Sand (g/kg)	11.40	0.0033	Ord2-Ord3; Ord2-Ord4
Total_Sand (g/kg)	13.32	0.0013	Ord2-Ord3; Ord2-Ord4
Silt (g/kg)	13.04	0.0015	Ord2-Ord3; Ord2-Ord4
Clay (g/Kg)	11.49	0.0032	Ord2-Ord3; Ord2-Ord4

Note:

Ord 2, 2nd-order tributary; Ord3, 3rd-order tributary; Ord4, 4th-order tributary.

**Table 4:** Spearman correlation between edaphic-topographic and hydrological parameters along the 21 plots.

Variables	Topographic		Hydrological	
	Elevation by GPS		Maximum river level (MRL)	
	(r)	(*p*-value)	(r)	(*p*-value)
Moisture	0.16	0.4644	−0.73	0.0002
pH	−0.04	0.888	−0.50	0.0215
Organic_Matter	−0.05	0.7755	−0.27	0.2456
P	−0.11	0.5804	0.12	0.5959
K	0.12	0.6138	−0.44	0.0488
Ca_Mg	0.24	0.2602	−0.5	0.0192
Al	−0.25	0.2767	0.65	0.0016
H_Al	−0.18	0.4785	0.45	0.0387
SNAC	0.23	0.2793	−0.5	0.0081
CEC	−0.20	0.4203	0.26	0.2641
Saturation_NAC	0.26	0.2555	−0.58	0.0056
Al_Saturation	−0.21	0.3362	0.68	0.0007
Clay	−0.28	0.2108	0.70	0.0004
Coarse_Sand	0.23	0.3133	−0.70	0.0004
Fine_Sand	0.01	0.9955	0.18	0.4385
Total_Sand	0.21	0.3362	−0.71	0.0003
Silt	−0.19	0.3878	0.62	0.0028
River tributary order	−0.37	0.1052	0.88	<0.0001
MRL	−0.47	0.0355	–	–

### Relationship between soil parameters and hydro-topographic gradients

**Figure 2:**
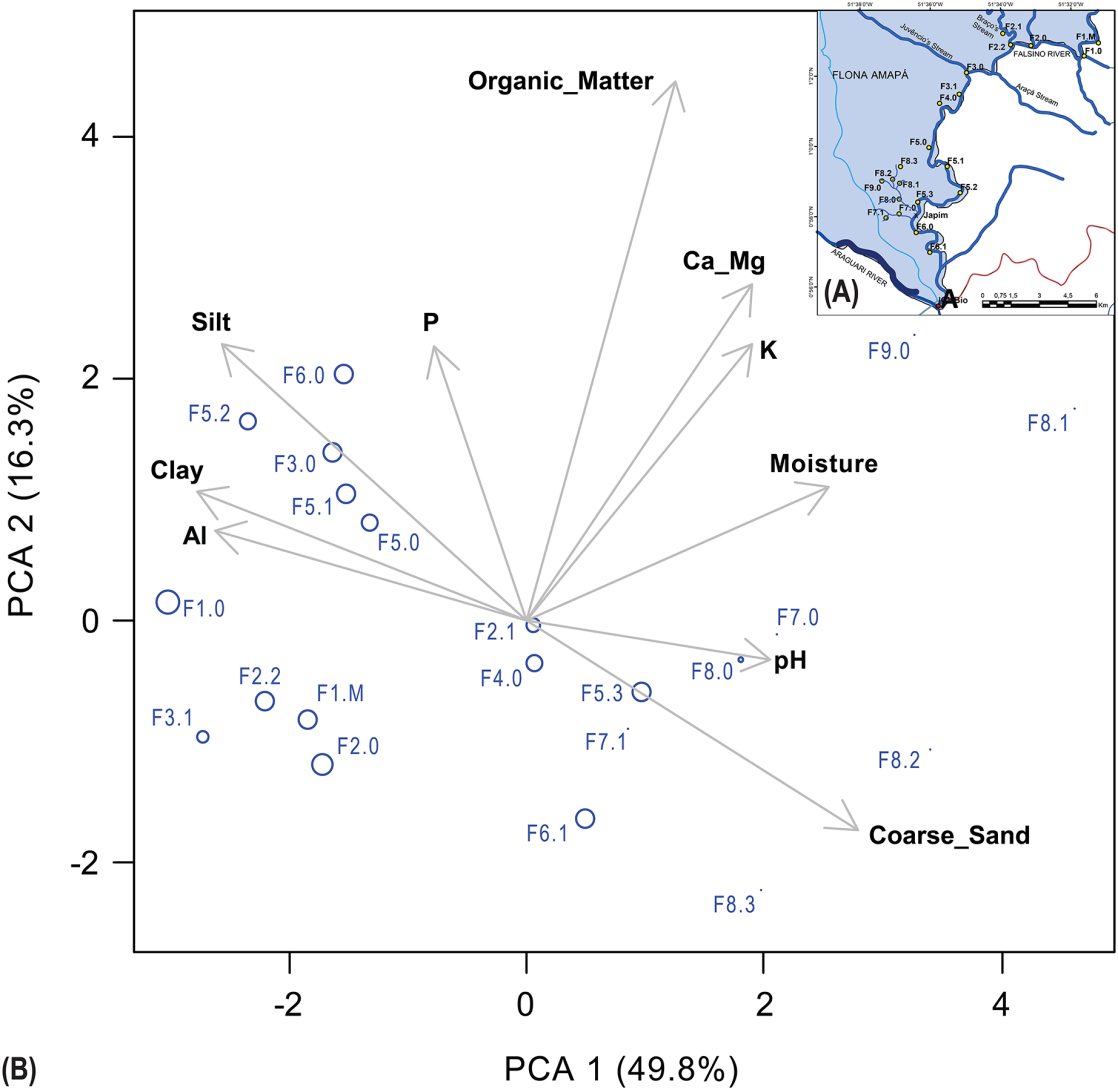
Principal Component Analysis (PCA) of edaphic variables in riparian zone plots. (A) Codification of riparian zone plots distributed in three spatial-topographic levels (Ord2, Ord2 and Ord4); (B) Principal Component Analyses of the edaphic variables. The size of the points is proportional to the Maximum River Level (MRL) in the plots.

To reduce the number of variables and visualize the integrated variations, the PCA test was applied. The PCA results are shown in Fig. 2.

The first two axes, or principal components, accounted for 49.8% (PCA1) and 16.3% (PCA2) of the variation in the edaphic attributes, totaling 66.1%. Theoretically, these axes are not correlated with each other. The first four principal components together represented 88.3% of the variability.

In the riparian plots of the Falsino River basin, the first soil component appears to capture a textural gradient, predominantly influenced by the variables coarse sand (+0.40), in opposition to clay (−0.40) and silt (−0.37). In addition to these variables, Al (−0.37) and moisture (+0.36) also contributed to PCA1, suggesting an interaction between texture and acidity.

For the second principal component (PCA2), the most significant contributions came from organic matter (+0.64), Ca_Mg (+0.40), K (+0.33), P (+0.32), and silt (+0.32). In other words, this component was more related to fertility (nutrients Ca_Mg, K, P), organic matter, and finer soil fractions.

In the PCA analysis, observing the arrangement of the plots according to the variation in edaphic attributes, the first principal component (PCA1) was associated with plots F8.1, F8.2, F9.0, and F8.0, located in the headwaters, which had the highest levels of the attributes most positively correlated with this component. In this case, coarse sand was positively correlated with PCA1. There were also parameters negatively correlated with this axis, such as aluminum and clay content. Thus, it was possible to observe, through the data and PCA analysis, low values of Aluminum, negatively correlated with PCA1, while the pH values were higher, positively correlated with PCA1, indicating less acidic soils with lower aluminum content.

These plots (F7 to F9) are located on the banks of second-order rivers with lower amplitudes and maximum flood levels. Furthermore, plots F7.0, F7.01, and F8.3 in this section also presented the lowest maximum river levels (MRL). As for the second principal component (PCA2), plots F6.1, F8.3, F8.2, F3.1 and F2.0 had the lowest values of organic matter. For example, aluminum concentration was high and moderately correlated with clay, silt, and coarse sand.

In addition to moderate correlations among edaphic parameters, there were significant moderate to strong correlations between edaphic parameters and MRL, see highlighted correlations in Table 4.

**Figure 3:**
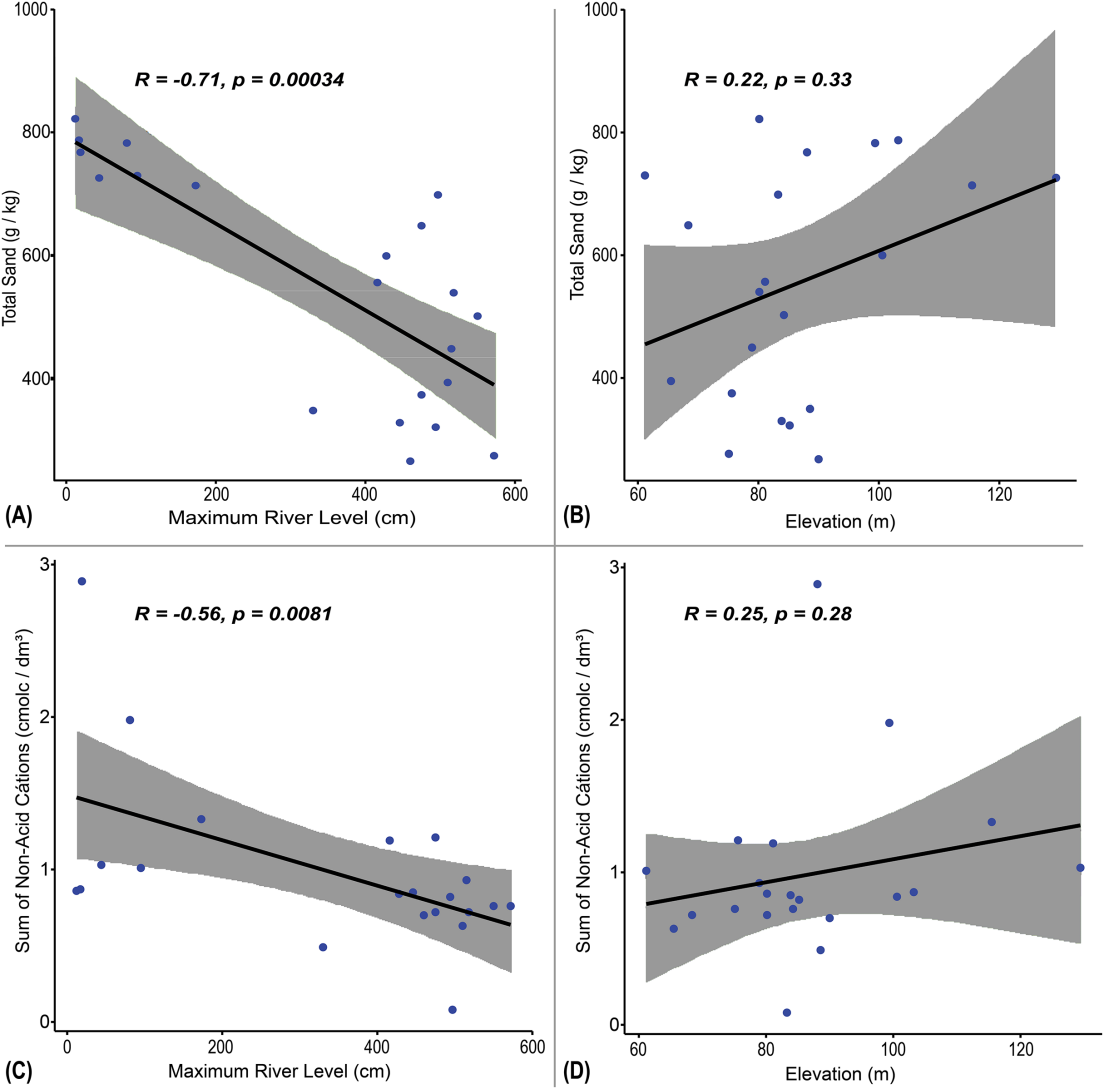
Spearman correlation tests between soil properties and hydrological/topographic variables in riparian plots. Spearman correlation tests between: (A and B) Total Sand concentration (g/kg); (C and D) Sum of Non-Acid Cations (cmol/dm^3^); in relation to Maximum River Level (MRL, cm) and Plot Elevation measured by GPS (m).

According to Fig. 3, the total sand concentration was inversely proportional to the MRL. This correlation also resulted in the best significance among all edaphic variables with MRL (r = −0.71, *p* = 0.00034) (Fig. 3A). On the other hand, the total sand concentration was positive with the elevation of the terrain (topography), but it was not significant (r = 0.22, *p* = 0.33) (Fig. 3B). Which may suggest that, in this case, there is a greater deposition of smaller sediments in the sites with higher MRL.

The Sum of Non-Acid Cations, which summarizes the attributes Ca_Mg and K, showed a moderate negative correlation with the MRL and high statistical significance, indicating a strong relationship with the evaluated hydrological variable (MRL) (r = −0.56, *p* = 0.0081) (Fig. 3C). On the other hand, the Sum of Non-Acid Cations was directly proportional to the elevation of the terrain (topography), but with a weak and non-significant correlation (r = 0.25, *p* = 0.28) (Fig. 3D).

In summary, all correlations between edaphic parameters and elevation were weak and not statistically significant (Table 4).

### Edaphic variations explained by elevation measurement methods (GPS and SRTM)

**Figure 4:**
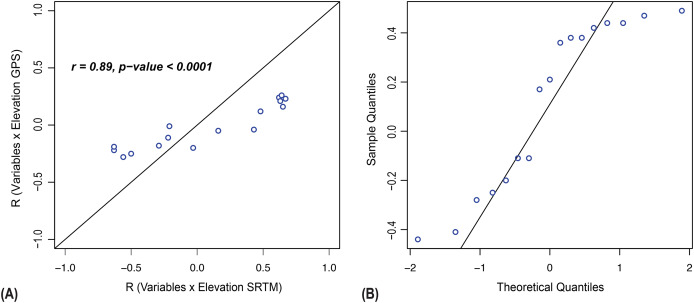
Statistical comparison of Spearman correlation results using GPS and SRTM elevation data. (A) Correlation analysis and (B) Wilcoxon test to evaluate the independence between Spearman correlation results obtained using topographic elevation data from GPS and from SRTM (Shuttle Radar Topography Mission).

For each variable, a pair of correlation coefficients (R values) were obtained: R_GPS_ (correlation between the variable and GPS-derived elevation) and R_SRTM_ (correlation between the variable and SRTM-derived elevation). Figure 4A displays these paired coefficients; each point represents the two values calculated for the same edaphic parameter. As expected for correlation measures, R_GPS_ and R_SRTM_ range between –1 and 1. Points lying closer to the 45-degree reference line indicate greater similarity between the results produced by the two methods.

The strong association between R_GPS_ and R_SRTM_ (r = 0.89, *p* < 0.0001) indicates that both elevation sources produce highly similar edaphic–topographic correlation patterns. The Q–Q plot of the paired differences (Fig. 4B) shows deviations from normality, supporting the use of the Wilcoxon paired-sample test. Consistent with the visual patterns in Fig. 4A, the Wilcoxon test detected no significant differences between R_GPS_ and R_SRTM_ (*p* > 0.05), confirming that the strength of edaphic–topographic correlations does not differ between the two elevation measurement methods.

## Discussion

This study offers new insights into the relationship between hydrological and edaphic factors in the riparian zones of the Falsino River basin in Eastern Amazonia. Through comprehensive analyses of topographic variation, flood pulse intensity, and detailed physicochemical assessments of soil properties, it deepens our understanding of the interactions shaping these environments. While the effects of flood pulses on soil characteristics are well documented, this study distinguishes itself by integrating GPS and SRTM derived topographic measurements to assess how basin elevation relates to MRL across tributary orders.

In addition to examining flood pulse effects, the study emphasizes spatial dynamics, revealing how MRL variation is influenced by both stream order and topographic gradients. This approach sheds light on the mechanisms driving soil heterogeneity in the complex geological and ecological context of the Amazon. These findings highlight the role of topographical and hydrological interactions in shaping biogeochemical patterns within riparian ecosystems, offering a more nuanced understanding of their functioning.

### Flood pulse in geographic and topographic gradients

The order of the water body is the factor that most influences the variation of the flood pulse in the Falsino River basin. Higher-order streams, located closer to the channel outlet, presented higher flood pulse levels, suggesting an accumulation effect along the longitudinal gradient of the drainage system. This supports the first hypothesis of the study, which predicted that flood pulse intensity would vary with both topography and stream order. This spatial pattern aligns with the observations of Wittmann et al. (2022), who describe the flood pulse as a predictable and high-amplitude hydrological phenomenon in Amazonian rivers, especially near basin mouths.

Despite the significant relationship observed, the high collinearity among edaphic variables (Fig. 2), limits the ability to isolate individual variables that may causally relate to flood pulse and soil physical-chemical properties. Nevertheless, the data confirm that both hydrographic hierarchy and topographic gradients jointly shape the flood pulse in riparian zones of this Amazonian sub-basin.

However, in many river basins, including parts of the Amazon, the construction of dams has significantly altered these natural regimes (Timpe & Kaplan, 2017), reducing the duration, magnitude, and predictability of floods. These disruptions affect the spatial and temporal heterogeneity of floodplain habitats and compromise the resilience of riparian systems (Zanon, 2021; Householder et al., 2021). Our results highlight the importance of considering flood pulse dynamics in ecological assessments of riparian environments, especially in Amazon regions where hydrological regulation is expanding.

Across the Falsino River basin and similar Amazonian sub-basins, multiple interrelated factors converge to shape riparian ecosystem dynamics. These factors include flood pulse variation associated with river order and topographic gradients, as well as anthropogenic influences such as dam-induced hydrological regulation and soil alteration. The resulting hydrological and edaphic disruptions can trigger cascading ecological effects on ecosystem processes, threatening the integrity of river-floodplain systems and potentially decoupling the synchrony of biological cycles with seasonal hydrological phenomena (Zanon, 2021; De Souza et al., 2024).

### Relationship between soil parameters and hydro-topographic gradients

The highly weathered soils of eastern and central Amazonia are the result of high temperatures, elevated humidity and prolonged leaching processes, which remove nutrients from the parent material and limits the contribution of the rocky base to soil fertility. Tributaries that drain these landscapes, such as those feeding the Falsino River, tend to carry low sediment loads and exhibit low nutrient concentrations, particularly in black- and clear-water rivers (Quesada et al., 2011; Wittmann et al., 2022).

These dark rivers typically originate in the Precambrian Guiana Shield, where white sand podzols dominate (Junk et al., 2015; Wittmann et al., 2022), and are characterized by water rich in dissolved organic matter, features that also define the Falsino River (Cunha et al., 2013; Da Silva et al., 2023; Sousa, Araújo & da Cunha, 2024; Santos & Cunha, 2015). Dark-water River basins typically drain sandy, nutrient-poor soils, which shape the chemical properties of their waters (Brazil, 2006; Junk et al., 2011).

Our findings are consistent with this hydrochemical and pedological context. Nutrient concentrations in the soils of the Falsino River basin were lower than those typically found in floodplains of the Central (Sioli, 1984) and Eastern Amazon (Ward et al., 2013; Santos et al., 2018b; Crizanto et al., 2024), where clay-rich soils and Andean sediment inputs contribute to higher fertility (Junk et al., 2015). These contrasts highlight the influence of regional geomorphology and water sources on edaphic development in riparian zones.

An edaphic survey by Oliveira et al. (2020), conducted in the southern portion of the FLONA/AP near riparian plots F7 to F9 (Fig. 1B), reports values closely aligned with those from our riparian plots for parameters such as sand content, pH, and exchangeable potassium. For example, they reported a mean pH of 4.56 ± 0.19 for the surface layer (0–40 cm), which is consistent with the strongly acidic conditions observed in our plots. In our study, pH was determined from composite samples of the 0–20 cm layer, and no significant variation among depths was detected.

The soils of the State of Amapá are generally acidic and frequently influenced by organic matter content (Alves, Alves & Mochiutti, 1992; De Souza et al., 2024). For instance, Oliveira (2012) reported mean organic matter concentrations of 21.1 ± 6.9 g/Kg in upland areas of FLONA/AP, which is lower than the mean value recorded in our riparian plots (43.41 ± 15.96 g/Kg). In the same study, potential acidity values (H_Al) were relatively high (10.07 ± 1.71 cmolc/dm^3^). It is important to note those were collected in non-flooded “terra firme” forest areas at higher elevations within the same conservation unit (Sousa, Araújo & da Cunha, 2024), which likely explains the differences in organic matter accumulation and acidification patterns.

In our study, total sand content consistently exceeded 50% on average across all riparian plots, ranging from 87% to 27%, from headwaters to mouth, significantly surpassing clay and silt fractions (Fig. 1A). In 13 of the 21 plots, the percentages of total sand fractions exceeded 50%. According to the Brazilian Soil Classification System (Santos et al., 2018a), these soils fall within the sandy loam textural subgroup, defined by a sand concentration above 520 g/kg. This pattern is supported by broaded regional surveys, which reported that most soils in the FLONA/AP are composed of lateritic mineral substrates rich in coarse fractions, ranging from 60% to 90% (ICMBio, 2016).

Total sand also showed a strong negative correlation with MRL (r = −0.71, *p* = 0.00034; Fig. 3A), suggesting that sandier soils are associated with less frequently flooded areas. This variable decreased in plots with higher flood pulse intensity (MRL), while clay and silt increased. Although topography showed no significant correlations with granulometric parameters, flood dynamics appear to be a stronger driver of soil texture variation in the Falsino basin. This aligns with Baniya et al. (2019), who reported that floodplains undergo physical and chemical changes after flood flows, including shifts in substrates texture from coarser to finer grains.

The total sand content was correlated with the mean flow velocities reported by Da Silva et al. (2023), based on measurements taken near the riparian plots, to explain the negative relationship observed between total sand and MRL. However, the data showed a weak and non-significant correlation between total sand and flow velocity, which may be attributed to the limited number of sampling points (*n* = 7). Moreover, the mean flow velocity was 1.20 m/s, considering only the sampling sites from Da Silva et al. (2023) corresponding to the riparian plots. This value indicates a flow velocity classified as moderate to high.

The significant correlation between MRL and several edaphic variables, including moisture, pH, exchangeable bases (Ca_Mg, K), potential acidity (H_Al), aluminum concentration, aluminum saturation, and soil texture fractions (clay, coarse sand, total sand, and silt), emphasizes the central role of flood dynamics in shaping riparian soils. No significant correlation was found with organic matter, P, CEC or fine sand, suggesting they may be regulated by processes independent of flood regime, such as vegetation or internal soil processes.

The headwaters of the Falsino River, located in the ancient Guyana shield, are characterized by limited pedogenic development due to highly weathered crystalline parent material. Although the Falsino basin lies on an old geological surface, its soils are generally less developed than those formed over reworked sediments in the Central Amazon (Quesada et al., 2010). The same occurs in the estuarine zones of the Eastern Amazon, where geomorphological conditions influence downstream geochemical profile (da Cunha & da Sternberg, 2018; Santos et al., 2018b; De Souza et al., 2024). In this predominantly sandy landscape, our findings suggest that flood-induced depositional processes gradually enrich the surface soils with finer particles such as silt and clay.

Decomposition under hypoxic conditions, typical of prolonged flooding in low-lying topographic areas, tends to be reduced, potentially affecting nutrient availability and organic matter content (Gallardo, 2003; Tsheboeng, Bonyongo & Murray-Hudson, 2017). Therefore, a positive relationship between flooding intensity and soil organic matter could be expected.

However, in our study, organic matter did not correlate significantly with either MRL (r = −0.27, *p* > 0.05) or elevation (r = −0.07, *p* > 0.05). When analyzed by depth (0–5, 5–10, and 10–20 cm), the correlations between organic matter and MRL remained weak and non-significant (r = −0.35 to 0.19, *p* > 0.05), as did those with elevation (r = –0.10 to 0, *p* > 0.05). These results indicate that, within the spatial scale sampled, organic matter content was not strongly structured by the hydro-topographic gradient. In contrast, higher MRL was associated with increased aluminum and acidity, while macronutrient levels (sum of non-acid cations) declined.

Nutrient exchange in floodplain environments such as igapó is shaped by seasonal flooding, which enables the transport of dissolved soil nutrients through lateral flow (Tockner & Stanford, 2002; Tsheboeng, Bonyongo & Murray-Hudson, 2017). These nutrients may derive from prior floods or organic matter decomposition during dry periods (Powell, 2009). Extensive drainage networks facilitate the long-distance transport of sediments, nutrients and propagules (Baniya et al., 2019; Da Silva et al., 2023; De Souza et al., 2024).

According to Baniya et al. (2019), increased river discharge can mobilize sediments and nutrients, influencing both transport rates and the connectivity between aquatic and terrestrial systems (Tockner & Stanford, 2002; da Cunha & da Sternberg, 2018; Santos et al., 2018b). Flow velocity and channel slope, analyzed by Cunha et al. (2013) in the Araguari and Falsino Rivers, affect areas near FLONA/AP, where stronger currents are associated with reduced plankton richness due to decreased retention time. The presence of hydroelectric plants along the Araguari River (Coaracy Nunes, Ferreira Gomes e Cachoeira Caldeirão) also influences the Falsino River’s hydrology by altering the downstream flow regime and nutrient availability in both soil and water (Santos & Cunha, 2015; Da Silva et al., 2023).

Small and medium-sized streams (1st to 5th order) comprise a substantial portion of the Amazon basin’s hydrological network and are often accompanied by floodplains that cover nearly 1 million km^2^ (Junk et al., 2015). In our study, we observed that lower-order tributaries of the Falsino River contribute notably to sediment and nutrient deposition in riparian soils, particularly in areas with higher flood pulse intensity. This aligns with (Cunha et al., 2013) who showed that flow dynamics in the Falsino River influence downstream hydrology, reinforcing the role of local tributaries in broader watershed processes. However, these ecosystems are highly vulnerable to disturbances, which can trigger ecological alterations and affect biotic communities (Schneider et al., 2017).

The flood pulse in the Falsino River basin influenced several edaphic attributes, partially supporting the second hypothesis of this study. However, elevationshowed non-significant correlation with any soil physical-chemical properties at the geographic scale analyzed. Recent studies, such as De Souza et al. (2024), provide complementary insights by showing that seasonal flooding in black-water floodplains structures tree community composition. Through floristic inventories and hydrological monitoring along the Falsino River, the authors found that prolonged and predictable flooding reduces taxon richness and phylogenetic diversity, promoting random lineage distribution. In contrast, “terra-firme” riparian forests exhibited greater taxonomic richness, phylogenetic dispersion, and endemism. These findings suggest that flooding acts as a selective filter for tree lineages, favoring traits adapted to root waterlogging and contributing to the diversification of Amazonian forests along hydro-edaphic gradients.

### Edaphic variations elucidated through GPS and SRTM quantification methods

Topographic data collected using GPS and SRTM showed a strong and consistent correlation in the Falsino River basin, supporting the third hypothesis. Despite differences in data acquisition, both methods produced comparable results in evaluating the relationship between elevation and edaphic parameters. Other studies using the Wilcoxon test have reported significant differences between methods, such as a mean elevation gap of 6 m (Kiser & Kelly, 2010) and 1.6-m in rugged terrains (Ahlgren, van Westrum & Shaw, 2024). The absence of such divergence in our study may reflect the moderate relief of the region and the spatial scale of analysis. These results emphasize the importance of evaluating terrain complexity when choosing elevation data sources, especially in studies that require high spatial accuracy.

One explanation for the strong correlation between GPS and SRTM data is that GPS technology, even in civilian and military applications, has been shown to be reliable for quantifying topographic parameters in the tropical forest regions with persistent cloud cover. This reliability makes GPS particularly useful for validating satellite-derived elevation data in field studies. Despite challenges such as dense vegetation and frequent rainfall, especially during the wet season, GPS remains effective in reducing measurement uncertainties and compensating for potential data gaps or distortions in satellite-based methods (Oliveira et al., 2020). This is especially valuable in remote Amazonian settings, where terrain accessibility is limited and accurate elevation estimates are essential for environmental assessments.

Although the SRTM model is known to produce underestimations in low-relief tropical landscapes (Kulp & Strauss, 2016), our results suggest that this limitation was not critical at the spatial scale examined and proved suitable for general topographic assessments in the Falsino basin. These findings support the use of SRTM as a cost-effective alternative for elevation modeling in remote Amazonian regions, where high-resolution methods, such as LiDAR or RTK-GPS are unfeasible (Zhang et al., 2019). While less precise than these high-resolution approaches, SRTM remains a valuable tool for capturing general topographic trends in conservation landscapes such as FLONA/AP. Together, these insights reinforce the methodological robustness of comparing GPS and SRTM data for evaluating hydro-edaphic interactions in complex tropical landscapes.

Although this study provides valuable insights into the relationships between topography, flood pulse, and the physicochemical attributes of soil in riparian zones, several limitations must be acknowledged. First, the spatial scale of the sampling design, although adequate for general characterization, may not fully capture the microscale heterogeneity typical of riparian zones in tropical environments. Second, the temporal resolution of flood monitoring (monthly over 1 year) may have missed short-term fluctuations or extreme events that affect flood pulse dynamics. Third, reliance on composite soil samples averaged by depth and location, while methodologically practical, may have masked localized variations in nutrient distribution and physical structure.

Additionally, the study focused mainly on abiotic variables, without integrating biotic components such as vegetation structure or land use, which are known to influence, and be influenced by, edaphic and hydrological gradients. Future studies integrating biotic variables and higher-resolution temporal and spatial sampling may yield a more comprehensive understanding of the eco-hydrological functioning of Amazonian riparian systems.

## Conclusions

This study demonstrated significant spatial variation in the flood pulse across the Falsino River basin, with maximum river level increasing consistently with stream order and decreasing elevation. Additionally, altimetry data obtained *via* GPS revealed a significant and moderate relationship (r = −0.47) between topography (elevation) and the flood pulse (MRL), reflecting the combined influence of hydrographic hierarchy and topographic position on hydrological behavior.

Flood pulse dynamics were shown to influence several edaphic properties, particularly soil texture and acidity. Soils in more frequently flooded areas exhibited higher clay and silt contents, along with increased aluminum concentration and potential acidity. In contrast, variables such as organic matter (humic substances), phosphorus, cation exchange capacity, and fine sand showed weak or no correlation with elevation or flood pulse, suggesting that these parameters may be influenced by additional ecological or biogeochemical processes. Moreover, GPS-derived elevation alone did not fully explain the variation in soil physicochemical properties.

The comparison between GPS and SRTM-derived elevation data revealed high consistency, indicating that both methods are reliable for assessing topographic variation in regions with moderate relief and restricted accessibility. These findings provide a practical framework for future hydropedological studies in tropical river basins, particularly in remote Amazonian landscapes.

Overall, the research highlights the interplay between topography, hydrology, and edaphic factors in riparian zones of the Eastern Amazon, offering new insights how these interdependent gradients structure riparian ecosystems and provides a methodological foundation for future landscape-scale assessments in similar tropical environments.

## Supplemental Information

10.7717/peerj.20882/supp-1 Supplemental Information 1 Values of the physicochemical parameters of each soil sample.

## Additional Information and Declarations

### Data Availability

The following information was supplied regarding data availability:

The raw data is available in the Supplemental File.
